# Herbivore-Associated Bacteria as Potential Mediators and Modifiers of Induced Plant Defense Against Spider Mites and Thrips

**DOI:** 10.3389/fpls.2018.01107

**Published:** 2018-07-30

**Authors:** Peter Schausberger

**Affiliations:** ^1^Department of Behavioural Biology, University of Vienna, Vienna, Austria; ^2^Sugadaira Research Station, Mountain Science Center, University of Tsukuba, Ueda, Japan

**Keywords:** induced plant response, bacteria, spider mites, thrips, endosymbionts, gut

## Abstract

Induced plant defense, comprising contact with exogenous stimuli, production of endogenous signals alerting the plant, associated biochemical cascades, and local and/or systemic expression of the defense mechanisms, critically depends on the nature of the inducing agents. At large, bio-trophic pathogenic microorganisms and viruses usually trigger the salicylate (SA)-mediated pathway, whereas necro-trophic pathogens and herbivores usually trigger the jasmonate (JA)-mediated pathway in plants. The SA- and JA-mediated pathways do not operate independently but commonly interfere with each other. Several recent studies revealed abnormal plant responses upon herbivore attack in diverse plant-herbivore systems. Observed abnormalities range from suppression of the common JA-pathway, induction of the SA-pathway to no response, yet the underlying proximate causes and ultimate consequences of these variations are elusive. Strikingly, some studies provide compelling evidence that anti-herbivore plant responses may decisively depend on bacteria associated with the herbivore attacking the plant (HAB for herbivore-associated bacteria). HAB may influence herbivore recognition by the plant and alter the biochemical cascades inside plants. Here, I report cases in point of HAB manipulating induced anti-herbivore plant responses, suggest spatial and temporal categorization of HAB, and point at proximate and ultimate aspects of plant defense manipulation by HAB. Following, I overview the diversity of HAB of spider mites and herbivorous thrips, argue that, considering recently reported phenomena of abnormal plant responses upon spider mite attack, some of these HAB could represent important, but hitherto largely neglected, mediators/modifiers of induced plant defense against spider mites and thrips, and conclude with suggestions for future research.

## Background

Plant defense against pathogenic and/or herbivorous organisms may be constitutive, induced or a combination of both. Induced plant defense comprises four phases: (i) contact with exogenous stimuli, (ii) production of endogenous signals alerting the plant, (iii) associated biochemical cascades, and (iv) local and/or systemic expression of the defense mechanisms. All phases critically depend on the nature of the inducing exogenous agents (Karban and Baldwin, 1997; Agrawal et al., 1999; Mithöfer and Boland, 2012). At large and from a generalizing/categorizing viewpoint, bio-trophic pathogenic microorganisms and viruses usually trigger the salicylate (SA)-mediated pathway, whereas necro-trophic pathogens and herbivores usually trigger the jasmonate (JA)-mediated pathway in plants. The SA- and JA-mediated pathways do not operate independently but influence, and commonly interfere with, each other (e.g., Thaler et al., 1999, 2012). Mechanistically, herbivore-attacked plants may defend themselves directly by changes in chemistry, such as the production of toxic substances (e.g., digestibility reducers) or proteinase inhibitors, and/or by morphological alterations, such as cell wall lignification, and/or indirectly by recruiting and fostering third trophic level natural enemies (predators and parasitoids) of the herbivores via emitting attractive volatiles or increasing the availability of alternative food. In detail and from a more sophisticated viewpoint, the above associations between defense-inducing agents and the SA- and JA-mediated pathways of plant defense are not that clear-cut. Induced plant responses to herbivores may vary between individuals, lines and populations of the same herbivore species. Observed abnormalities in plant response to herbivore attack range from suppression of the common JA-pathway, induction of the SA-pathway to no response, yet the underlying proximate causes of these variations and ultimate consequences for multi-trophic plant–herbivore–carnivore interactions are elusive. Strikingly, a number of recent studies provide compelling evidence that the response of plants to herbivore attack may be decisively influenced by microorganisms associated with the herbivore attacking the plant. Suggestions of herbivore-associated microorganisms possibly playing important roles in plant–herbivore interactions have been made decades ago (Berenbaum, 1988; Barbosa et al., 1991), yet rigorous experimental assessment has gained momentum only recently. Notably, studies on beetle larvae and whiteflies show that herbivore-associated microorganisms may fundamentally change the plant response to herbivore attack (Chung et al., 2013a,b; Su et al., 2015). Microorganisms playing decisive roles in induced plant defense may also be true for spider mites (Tetranychidae) and herbivorous thrips (Thripidae), many species of which are globally distributed and tremendously important as both agricultural pests and model organisms in plant-animal interaction research. These two families include two of the most destructive agricultural plant pests worldwide, i.e., the two-spotted spider mite *Tetranychus urticae* and western flower thrips *Frankliniella occidentalis*. Both species are highly polyphagous with >1000 host plant species reported for *T. urticae* (Bolland et al., 1998) and >250 host plant species reported for *F. occidentalis* (CABI, 2018). Spider mites feed on their host plants by piercing parenchyma cells and sucking out the cell contents; thrips feed on their host plants by rasping open the epidermal cells and sucking out the cell contents; thrips are also feared vectors of plant-pathogenic viruses (Rotenberg et al., 2015). Species of both families may be associated with a host of microorganisms, and phenomena akin to those observed in beetles and whiteflies, such as specific individuals, lines or populations eliciting abnormal plant responses upon attack, have been described. For example, Abe et al. (2012) observed a beneficial effect of tomato spotted wilt virus-infection in *Arabidopsis* on *F. occidentalis*’ performance by upregulating SA-mediated defense interfering with JA-mediated pathways. Kant et al. (2008) observed variation regarding JA-mediated defense induction and susceptibility in *T. urticae* on tomato *Lycopersicon esculentum*, among others with one non-inducing but still susceptible line; Sarmento et al. (2011) and Alba et al. (2015) described a line of *Tetranychus evansi* (an invasive spider mite species primarily occurring on solanaceous host plants) that suppresses both SA- and JA-mediated defense pathways in tomato. Whether or not microorganisms are at play in the spider mites under investigation has been largely untested (but see Staudacher et al., 2017).

This article focuses on potential implications of herbivore-associated microorganisms in modulation of plant responses induced by spider mites (Tetranychidae) and thrips (Thripidae). Due to the lack of knowledge for herbivore-associated viruses and fungi, except for transmission of plant-pathogenic viruses by various thrips species (Rotenberg et al., 2015) and the spider mite *Petrobia latens* (Robertson and Carroll, 1988), this article is primarily concerned with gut/saliva bacteria and facultative intracellular endosymbionts (i.e., secondary symbionts). Primary symbionts influencing herbivore–plant interactions, such as *Buchnera* sp. (see Engel and Moran, 2013 for review), are left out for no primary symbionts have been described in spider mites and thrips. For up-to-date treatises of diverse phenomena of microorganisms affecting plant–herbivore interactions across herbivore taxa, see Frago et al. (2012); Hansen and Moran (2014), Giron et al. (2017), and Shikano et al. (2017). The aims of this article are reporting cases in point of herbivore-associated bacteria (HAB) fundamentally changing induced plant defense against herbivores, overviewing the diversity of bacteria associated with spider mites and thrips, discussing possible roles of those bacteria in mediating induced plant defense against herbivores, and providing suggestions for pertinent future research using spider mites and thrips.

## Categories of Bacteria Potentially Mediating Plant Response to Herbivore Attack

**FIGURE 1:**
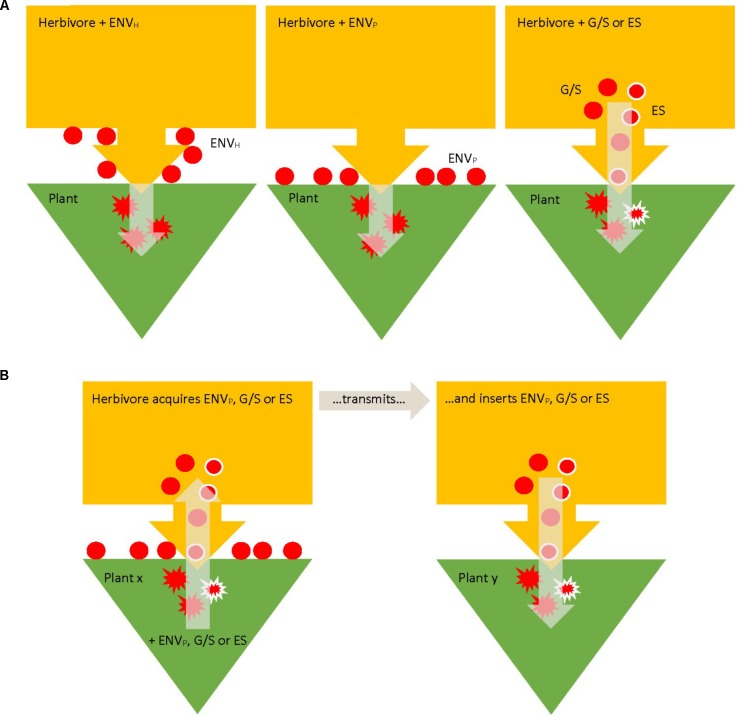
Pathways of transmission of herbivore (yellow)-associated bacteria (HAB; red) to their host plants (green). HAB may be environmental (ENV_H,P_; on the outside of the herbivore or plant), in the gut/saliva (G/S), or intracellular endosymbionts (ES; red with white margin), based on their location relative to the herbivore body. Circles symbolize HAB possibly involved in induced plant defense; splashes symbolize HAB activity in induced plant defense inside plant tissue. HAB may be intrinsically associated with the herbivores, either at the herbivore/plant interface or inside the herbivores **(A)** or may be acquired from plants and transmitted to other plants **(B)**.

Bacteria mediating the plant response to herbivore attack can be allocated to three, mutually non-exclusive, major groups, based on their location relative to the herbivore body (Hansen and Moran, 2014): environmental (or external), digestive system (internal extracellular), and endosymbionts (internal intracellular) (**Figure 1A**). Environmental (external) bacteria may reside on the outside of the herbivore, on the mouthparts or other body parts, or on the plant surface (Redford et al., 2010). Regarding environmental microorganisms much more is known about fungi than bacteria (Hansen and Moran, 2014) and experimental studies addressing the functions of environmental bacteria in plant–herbivore interactions are rare. Internal extracellular are mainly bacteria in the digestive system, i.e., in the gut and/or in the salivary glands (Dillon and Dillon, 2004; Engel and Moran, 2013); internal intracellular are mainly reproductive and other endosymbionts (Werren, 1997; Weeks et al., 2003; Engelstädter and Hurst, 2009). In any case, bacteria of all three location categories can be transmitted from herbivores to inner plant tissues by feeding activity (albeit with varying likelihood and reliability) and have the potential to change the defensive response of the plant to the herbivores (**Figure 1A**). HAB may further be allocated along the continuum from permanence to transience regarding their association with the herbivores. On the close end of this continuum are endosymbionts and bacteria in the digestive system reliably transmitted vertically or horizontally among conspecific individuals; on the distant end of this continuum are plant pathogens or other miscellaneous microorganisms present in the phyllosphere, which are just vectored by feeding activity from the outside to the inside of the plant or from one plant to another. Bacteria in the gut and/or salivary glands and intracellular endosymbionts influencing plant response may be transient if picked up from one plant by feeding and inserted or deposited in feces on another plant (**Figure 1B**), such as plant-pathogenic bacteria transmitted by spider mites (Choi et al., 2016) or may be permanently, or close to permanently, associated with herbivores such as secondary extra- and intracellular endosymbionts. In any case, acquisition of bacteria from plants may also lead to novel more permanent individual associations such as described for plant-mediated transmission of secondary endosymbionts (Caspi-Fluger et al., 2012 for *Rickettsia*; Gonella et al., 2015 for *Cardinium*; Li et al., 2017 for *Wolbachia*).

## Evidence of Hab Mediating Plant Response to Herbivore Attack

As in animals in general, herbivorous mites and insects house a host of different microorganisms in their gut and salivary glands, with the bacteriomes often being dominated by a small number of species, but high inter-taxon variability (Engel and Moran, 2013; Jones et al., 2013). Feeding herbivores commonly transmit gut/saliva bacteria to the plant. For example, Bansal et al. (2011) determined that up to 70% of the bacterial genera found in Hessian fly larvae (*Mayetiola destructor*) were also found in fly-infested wheat, indicating that the bacteria are transmitted to the host plant via feeding. Striking examples of gut/saliva HAB manipulating plant response come from Colorado potato beetle, *Leptinotarsa decemlineata* on tomato, *L. esculentum* (Chung et al., 2013a,b). Bacteria such as *Enterobacter*, *Stenotrophomonas*, and *Pseudomonas* in the beetle saliva induce plant defense against pathogens, i.e., the SA-dependent pathway, which in turn downregulates the JA-dependent pathway due to negative cross-talk (Thaler et al., 1999, 2012), altogether promoting growth of the beetle larvae (Chung et al., 2013a,b). Similar suppression of JA-mediated plant defense occurs in various *Solanum* sp. although different bacterial communities in the beetle saliva are involved (Chung et al., 2017). Also, in the false potato beetle *Leptinotarsa juncta*, bacteria in the regurgitate may suppress anti-herbivore plant defense in tomato and horsenettle, with host-plant-specific bacterial effects (Wang et al., 2016). Host plant specificity has also been shown for bacteria (e.g., *Pantoea*) in the saliva of fall armyworms *Spodoptera frugiperda*, which suppress anti-herbivore defense in tomato, but induce such defense in maize (Acevedo et al., 2017). In *Arabidopsis*, greenhouse whiteflies *Trialeurodes vaporariorum* induce the SA-dependent pathway but suppress JA-dependent anti-herbivore defense (Zarate et al., 2007); however, it is as yet unknown whether this is due to herbivore-associated microorganisms or intrinsic (endogenous) herbivore traits.

Tobacco whiteflies *Bemisia tabaci* harboring the facultatively endosymbiotic bacteria *Hamiltonella defensa* in their saliva suppress JA-dependent defenses compared to whiteflies free of the bacteria (Su et al., 2015). In the tomato psyllid, *Bactericerca cockerelli*, the endosymbiont Candidatus Liberibacter psyllaurous (which can also be plant-pathogenic) downregulates both JA- and SA-dependent defense responses of tomato plants (Casteel et al., 2012). *Wolbachia*, *Cardinium*, and *Spiroplasma* are widespread, primarily maternally transmitted symbionts manipulating reproduction in many arthropods (for review, see Engelstädter and Hurst, 2009). Among the secondary symbionts, *Wolbachia* is the most widely documented and the most prominent reproductive endosymbiont, infecting a large proportion of arthropod species (Werren et al., 2008). *Wolbachia* is best known for reproductive manipulation of their hosts to enhance its own spread while lowering host fitness. Recent studies provide a growing body of evidence for *Wolbachia*-associated fitness benefits (for review, see Zug and Hammerstein, 2015). Also, other bacterial endosymbionts, such as *Cardinium* (Weeks et al., 2003), *Spiroplasma* (Cisak et al., 2015), or *Rickettsia* (Weinert et al., 2009), ranked at decreasing prevalence in arthropods (Duron et al., 2010), are similarly able to manipulate reproduction of their hosts (Engelstädter and Hurst, 2009). These endosymbionts have been previously thought to be primarily vertically transmitted from mother to offspring but recent experimental evidence suggests that horizontal transmission via feeding on prey/hosts (Le Clec’h et al., 2013; Ahmed et al., 2015), mating (Moran and Dunbar, 2006), sharing hosts (Huigens et al., 2004; Duron et al., 2010) and/or feeding on plants (Caspi-Fluger et al., 2012 for *Rickettsia*; Gonella et al., 2015 for *Cardinium*; Li et al., 2017 for *Wolbachia*) is probably more common than previously anticipated. *Wolbachia* has been shown to persist in plant tissue for more than 50 days at unknown temperature (Li et al., 2017). *Wolbachia* is often also present in the salivary glands of their hosts (Dobson et al., 1999), where it may change the composition of the saliva, which in turn may influence plant defense. Contrasting evidence exists for *Wolbachia* mediating induced plant defense against larvae of the western corn rootworm *Diabrotica virgifera virgifera* in maize. While Barr et al. (2010) concluded that *Wolbachia*-infected but not uninfected weevil larvae suppress anti-herbivore defense in maize roots, Robert et al. (2013) did not find any evidence for differences in the plant response to *Wolbachia*-infected and uninfected weevil larvae. Possible reasons for these contrasting results include antibiotic treatments likely removing other microorganisms, including gut/saliva bacteria, from the weevils and/or the bacteriomes differing between the weevils used by Barr et al. (2010) and Robert et al. (2013).

## Proximate and Ultimate Considerations

Proximate questions are whether the substances abnormally modulating plant defense are produced by the herbivores themselves (and thus represent herbivore-associated-molecular patterns, HAMPs) or constitute metabolites of the bacteria in their saliva, the regurgitate or on the outside of their mouthparts (and thus represent microbe-associated-molecular patterns, MAMPs) (Maffei et al., 2012). Alternative underlying mechanisms include substances produced by the bacteria and the herbivores masking each other, or the herbivores are lacking substances that are normally needed by the plants to recognize the herbivores, such as linolenic acid in caterpillars of *Heliothis subflexa* (De Moraes and Mescher, 2014). HAB and/or their products may have direct effects on the plants or indirect via affecting (inducing or changing) the expression of elicitors, effectors or HAMPs in the herbivores’ saliva or regurgitate. For example, gut bacteria induce the expression of an elicitor in *Helicoverpa zea* triggering JA-dependent plant defense in tomato (Wang et al., 2017). In contrast, Acevedo et al. (2017) found that *Pantoea* did change the plant response to fall armyworms but did not change the expression of salivary proteins or known elicitors in the caterpillar’s saliva, indicating MAMPs. Abnormal plant responses upon herbivore attack could simply constitute recognition errors by the plant.

One may argue that herbivores evolved associations with specific microorganisms to disguise themselves from proper recognition by the plants and thus trick plants into activating biochemical cascades and defense against non-existent threats usually posed by pathogenic microorganisms. However, important ultimate questions to be addressed case by case are whether these phenomena truly represent counter-adaptations in the arms race between plants and herbivores targeted to disguise herbivore identity, deceive plants into incorrect recognition, and/or switch off or circumvent the defense response against herbivores, or whether it is side effects of accidental associations with microorganisms, or epiphenomena of co-evolved associations for other reasons than manipulating plant defense, or whether it is simply recognition errors by the plant. Answering those questions requires additionally assessing both direct and indirect fitness consequences of bacterial associates for the herbivores and considering the transience-permanence continuum of the bacteria–herbivore association. HAB providing direct fitness benefits to their host (Zug and Hammerstein, 2015) and additionally manipulating plant defense to the benefit of their hosts and promoting their own spread are clear indications of bacteria-herbivore co-evolution. However, even HAB lowering herbivore fitness in a direct way, may indirectly promote herbivore fitness if suppressing anti-herbivore plant defenses. Herbivores may yield net fitness benefits through provision of enemy-free space – because attacked plants do not cry for help the herbivores remain cryptic to their natural enemies (see De Moraes and Mescher, 2014) – or through the prevention of production of digestion inhibitors or other toxins (Boone et al., 2013; Douglas, 2013).

## Suspicious Bacteria Associated With Spider Mites and Thrips

Overall, regarding gut/saliva and intracellular endosymbiotic bacteria more studies are available for spider mites than thrips. Accordingly, there is a much wider spectrum and diversity of bacteria reported for spider mites than thrips (Supplementary Tables S1, S2) but this does not necessarily mean that spider mites are more prone to harbor bacteria than thrips because of differences in detection efforts. In any case, members of both families may harbor bacteria that have been found to manipulate plant defense in other arthropods (Supplementary Tables S1, S2). Among Tetranychidae, two-spotted spider mites *T. urticae* and *T. evansi* are especially well studied, with a large number and diversity of gut/saliva and endosymbiotic bacteria (e.g., Yoon et al., 2010; Knegt et al., 2017; Staudacher et al., 2017 for gut bacteria; e.g., Zélé et al., 2018 for endosymbionts). Spider mite species are especially likely to harbor single or multiple reproductive symbionts such as *Wolbachia*, *Cardinium*, or *Spiroplasma* (Supplementary Tables S1, S2). Notably, Staudacher et al. (2017) detected *Wolbachia* and *Cardinium* in *T. urticae* and Zélé et al. (2018) in *T. evansi*, lines of which have been described to abnormally manipulate plant defense against herbivores (Sarmento et al., 2011; Alba et al., 2015). The two most prominent thrips pests, western flower thrips *F. occidentalis* and onion thrips *Thrips tabaci* do not harbor reproductive endosymbionts but are stably associated with Enterobacteriaceae such as *Erwinia* and *Pantoea* (de Vries et al., 2001, 2008; Chanbusarakum and Ullman, 2008; Facey et al., 2015; Dutta et al., 2016a). Endosymbionts such as *Wolbachia* or *Cardinium* have been found in many other herbivorous thrips species such as *Thrips palmi* (Saurav et al., 2016). Virtually nothing is known about fungi, fungal spores, or fungal metabolites in the saliva of thrips and mites.

## Mind the Bacteria of Spider Mites and Thrips in Induced Plant Defense

Similar to other herbivores, for spider mites, there exist a number of studies reporting unusual plant responses upon attack (suppression of JA-mediated defense and/or induction of the SA-mediated pathway) (e.g., Kant et al., 2008; Sarmento et al., 2011; Alba et al., 2015). However, studies aiming at deciphering and suggesting potential involvement of microorganisms in modulation of abnormal plant responses are scarce and restricted to two-spotted spider mites *T. urticae* (Ueda et al., 2010; Staudacher et al., 2017). Both studies report that removal of microorganisms from the spider mites by antibiotics changes the response of tomato and bean plants to their attack. Most notably, among other differences in plant response induction among doubly infected, singly-infected and non-infected mites, Staudacher et al. (2017) observed downregulation of JA-precursors but upregulation of SA following removal of *Wolbachia* but conservation of *Spiroplasma*. Some lines of *T. evansi* and *T. urticae* may suppress both SA- and JA-dependent plant defenses (Sarmento et al., 2011; Alba et al., 2015), which has been associated with altered expression of genes responsible for the production of effector proteins in the spider mite saliva (Jonckheere et al., 2016; Villarroel et al., 2016). Nonetheless, HAB influence and altered gene expression are rather inter-related than mutually exclusive explanations for induction of abnormal plant responses. HAB may both influence gene expression of their hosts, as documented in the fruit fly *Drosophila melanogaster* (Broderick et al., 2014; Combe et al., 2014), and alter the occurrence and composition of salivary proteins, as documented for *Wolbachia* and *Spiroplasma* in the spider mite *Tetranychus truncatus* (Zhu et al., 2018). Abnormal plant responses could also be due to HAB influence in the evolutionary past if genes coding for enzymes involved in mite-plant interactions stem from horizontal gene transfers between HAB and spider mites (Wybouw et al., 2018). Additional or alternative explanations on the plant side include multiple signals masking each other or reflecting a conflict in the response of the plant to herbivore attack because of simultaneously perceiving endogenous spider mite signals (normally inducing the JA pathway) and bacteria-related signals (normally inducing the SA pathway), together resulting in no or suppressed plant response. Overall, it cannot be excluded that intraspecific variations in induction of plant responses observed in different spider mite species such as *Tetranychus kanzawai* (Matsushima et al., 2006), *T. evansi* (Sarmento et al., 2011), and *T. urticae* (Kant et al., 2008), which correlate with genetic differences (Yano et al., 2003; Villarroel et al., 2016), may be co-determined by differences in the bacteriome (Yoon et al., 2010; Knegt et al., 2017; Zélé et al., 2018). Genetic intraspecific differences such as resistance to acaricides may covary with differences in the quantity and diversity of HAB (Yoon et al., 2010). In addition to reproductive endosymbionts, Yoon et al. (2010) detected also *Pantoea*, *Enterobacter*, and *Pseudomonas* in the gut of *T. urticae*; in the absence of reproductive endosymbionts, Knegt et al. (2017) detected *Pseudomonas* and *Stenotrophomonas* in the gut of *T. evansi*. These gut bacteria have potential to change and manipulate anti-herbivore plant response, as shown for Colorado potato beetles (Chung et al., 2013a,b), false potato beetles (Wang et al., 2016), and fall armyworms (Acevedo et al., 2017).

Compared to spider mites, only little is known about induced plant responses to thrips attack (for review, see Steenbergen et al., 2018), not to speak of plant responses manipulated by thrips-associated bacteria. Thrips are well known for vectoring plant-pathogenic viruses such as tospoviruses (Rotenberg et al., 2015). Abe et al. (2012) observed that virus transmission may alter the anti-herbivore plant response to the benefit of thrips, i.e., upregulate the SA-dependent pathway and downregulate the JA-dependent pathway. This trade-off is unsurprising and readily comprehensible given that the plant must simultaneously deal with two types of threats. Nonetheless, even the few gut and endosymbiotic bacteria found in association with herbivorous thrips (Supplementary Tables S1, S2) indicate potential for manipulation of anti-thrips plant response. For example, western flower thrips and onion thrips are associated with *Erwinia* (de Vries et al., 2001, 2008) and *Pantoea* or *Pantoea*-like bacteria (Facey et al., 2015; Dutta et al., 2016a), which could influence anti-herbivore plant responses. Dutta et al. (2016b) reported changes in plant response to thrips *Frankliniella fusca* upon deposition of feces containing *Pantoea ananatis* on the plant surface but not upon contact with salivary secretions.

## Conclusions and Suggestions for Future Research

Given the widespread occurrence and diversity of gut/saliva and endosymbiotic bacteria found in spider mites and thrips, which have been shown for their ability to manipulate anti-herbivore plant defenses in other arthropod taxa, it is more than plausible to anticipate that these bacteria play a role in anti-spider mite and/or anti-thrips plant response. Current methodological standard is comparing the plant response to untreated herbivores harboring microorganisms to that of herbivores treated with broad spectrum antibiotics, such as tetracycline, and thus being free, or below detectability, or having strongly reduced titers, of bacteria. However, broad spectrum antibiotic treatment eliminates or reduces both gut/saliva and endosymbiotic bacteria in the herbivores or changes the bacterial composition due to varying susceptibility (see Staudacher et al., 2017). Thus, broad spectrum antibiotic treatments may blur cause and effect of HAB. Studies comparing the response to herbivores treated with broad spectrum antibiotics and left untreated as the only experimental way of assessing the effect of HAB remain correlational, but do not provide stringent proof of cause and effect. Such studies are important and certainly needed to obtain indications of microorganisms at play but do not allow concluding on one species or operational taxonomic unit (OTU) of eliminated/changed microorganism to be the causative agent of an observed effect. Use of antibiotics is also suitable to disentangle expression of HAB-transferred genes (Wybouw et al., 2018) versus endogenous HAB genes. In an ideal case, future studies should strive to achieve selective elimination of single bacteria taxa, to pinpoint which bacteria are responsible for manipulating plant defense. If possible, supplemental experiments should examine plant responses to cultured OTUs and their metabolites and test for masking, synergism, or interference among MAMPs and HAMPs, elicitors, and effectors, in the saliva and regurgitates of herbivores. Multiple bacterial infections may turn negative direct effects of each bacterial taxon alone into direct positive fitness effects for their host (see Zhang et al., 2018 for *T. truncatus* doubly infected with *Wolbachia* and *Spiroplasma*); the same may apply to the direct effects of multiple versus single HAB on induced plant response (Staudacher et al., 2017), indirectly affecting fitness of the herbivores. Clearly, investigating the role of HAB of spider mites and thrips in induced plant defense will provide a highly exciting, promising and fruitful avenue of future research.

## Author Contributions

PS confirms being the sole contributor of this work and approved it for publication.

## Conflict of Interest Statement

The author declares that the research was conducted in the absence of any commercial or financial relationships that could be construed as a potential conflict of interest.
