# Supplementary material for: Herbivore-Associated Bacteria as Potential Mediators and Modifiers of Induced Plant Defense Against Spider Mites and Thrips
Source: Front Plant Sci. 2018 Jul 30;9:1107. doi: 10.3389/fpls.2018.01107 (PMC6077224; doi:10.3389/fpls.2018.01107)
Supplement: Supplementary file 1 [file Table_1.pdf]

Table S1. Endosymbiotic and gut/saliva bacteria documented in species of the spider mite family Tetranychidae (Acari, Trombidiformes) and the thrips family Thripidae (Insecta, Thysanoptera). References are not meant to be exhaustive.

**Herbivore**	**Bacteria** Class	Genus/species	**Localization**[1]	**References**[2,3]
**Tetranychidae**				
Tetranychus urticae	α-Proteobacteria	*Wolbachia*	ES	a,b,c,e,f,g,h,i,k,m,n, o,p,q,t,u,v,w,x,ab,aj,am
	Bacteroidetes	*Cardinium*	ES	h,i,j,k,o,q,r,s,x,ab,ai,aj
	Mollicutes	*Spiroplasma*	ES	o,aj
	α-Proteobacteria	*Caulobacter*	ES	m
	α-Proteobacteria	*Rickettsia*	ES	m,ab,am
	γ-Proteobacteria	*Enterobacter*	G/S	v
	γ-Proteobacteria	*Pantoea*	G/S	v
	Bacilli	*Bacillus*	G/S	v
	γ-Proteobacteria	*Pseudomonas*	G/S	v
	α-Proteobacteria	*Sphingomonas*	G/S	v
	β-Proteobacteria	*Herbaspirillum*	G/S	v
	Bacilli	*Staphylococcus*	G/S	v
	γ-Proteobacteria	*Pasteurella*	G/S	v
	γ-Proteobacteria	*Citrobacter*	G/S	v
	β-Proteobacteria	*Acidovorax*	G/S	v,af
	Flavobacteria	*Chryseobacterium*	G/S	v
	β-Proteobacteria	*Janthinobacterium*	G/S	v
	β-Proteobacteria	*Xenophilus*	G/S	v
	101 OTUs in 33 families; mainly γ-Proteob.		G/S	aj
	(Enterobacteriaceae, Pseudomonaceae)		G/S	aj
T. evansi	α-Proteobacteria	*Wolbachia*	ES	am
	Bacteroidetes	*Cardinium*	ES	am
	α-Proteobacteria	*Rickettsia*	ES	am
	228 OTUs; mainly γ-Proteobacteria incl.		G/S	ah
	Pseudomonas and *Stenotrophomonas*		G/S	ah
T. ludeni	α-Proteobacteria	*Wolbachia*	ES	am
	Bacteroidetes	*Cardinium*	ES	am
T. truncatus	α-Proteobacteria	*Wolbachia*	ES	q,y,aa,ab,ac,ae
	Bacteroidetes	*Cardinium*	ES	y,ab,ae
	Mollicutes	*Spiroplasma*	ES	y,ab,ac
T. kanzawai	α-Proteobacteria	*Wolbachia*	ES	a,g,aa,ab
	Bacteroidetes	*Cardinium*	ES	ab
T. pueraricola	α-Proteobacteria	*Wolbachia*	ES	g,h,aa,ab
	Bacteroidetes	*Cardinium*	ES	h,r
T. phaselus	α-Proteobacteria	*Wolbachia*	ES	aa,ab,ad
	Bacteroidetes	*Cardinium*	ES	ab,ad
T. yusti	α-Proteobacteria	*Wolbachia*	ES	a
T. neocaledonicus	α-Proteobacteria	*Wolbachia*	ES	a
T. piercei	α-Proteobacteria	*Wolbachia*	ES	x,ab
	Bacteroidetes	*Cardinium*	ES	x,ab
T. turkestani	α-Proteobacteria	*Wolbachia*	ES	a,c
T. parakanzawai	α-Proteobacteria	*Wolbachia*	ES	g
Amphitetranychus viennensis	α-Proteobacteria	*Wolbachia*	ES	q,ab

	Bacteroidetes	*Cardinium*	ES	[q,ab]
A. quercivorus	Bacteroidetes	*Cardinium*	ES	[h,r]
Bryobia rubrioculus	α-Proteobacteria	*Wolbachia*	ES	[d,p,u]
	Bacteroidetes	*Cardinium*	ES	[p,u]
B. praetiosa	α-Proteobacteria	*Wolbachia*	ES	[d,t,u]
B. kissophila	α-Proteobacteria	*Wolbachia*	ES	[d,u]
B. graminum	α-Proteobacteria	*Wolbachia*	ES	[d]
B. neopraetiosa	α-Proteobacteria	*Wolbachia*	ES	[d]
B. sarothamni	α-Proteobacteria	*Wolbachia*	ES	[s,t,u]
	Bacteroidetes	*Cardinium*	ES	[s,t,u]
B. berlesei	α-Proteobacteria	*Wolbachia*	ES	[u]
Eotetranychus suginamensis	Bacteroidetes	*Cardinium*	ES	[h,r]
E. uncatus	Bacteroidetes	*Cardinium*	ES	[p]
Eutetranychus orientalis	α-Proteobacteria	*Wolbachia*	ES	[a]
Oligonychus gotohi	α-Proteobacteria	*Wolbachia*	ES	[g]
O. ilicis	Bacteroidetes	*Cardinium*	ES	[h,r]
O. biharensis	α-Proteobacteria	*Wolbachia*	ES	[a]
O. ununguis	α-Proteobacteria	*Methylobacterium*	G/S	[ag]
	Bacilli	*Bacillus thuringiensis*	G/S	[ag]
	Bacilli	*Bacillus cereus*	G/S	[ag]
	Bacilli	*Staphyloc. chromogenes*	G/S	[ag]
	γ-Proteobacteria	*Pseudomonas oleovorans*	G/S	[ag]
	Actinobacteria	*Streptomyces*	G/S	[ag]
	Bacilli	*Paenibacillus apiarus*	G/S	[ag]
	Actinobacteria	*Mycobacterium*	G/S	[ag]
Panonychus mori	α-Proteobacteria	*Wolbachia*	ES	[g]
P. ulmi	Bacteroidetes	*Cardinium*	ES	[z]
Petrobia harti	α-Proteobacteria	*Wolbachia*	ES	[i,ab]
	Bacteroidetes	*Cardinium*	ES	[i,u,ab]
Schizotetranychus cercidiphylli	α-Proteobacteria	*Wolbachia*	ES	[g]
Stigmaeopsis celarius	Bacteroidetes	*Cardinium*	ES	[r,ai]
S. longus	α-Proteobacteria	*Wolbachia*	ES	[ai]
	Bacteroidetes	*Cardinium*	ES	[r,ai]
S. miscanthi	Bacteroidetes	*Cardinium*	ES	[ak]
	Mollicutes	*Spiroplasma*	ES	[ak]
S. nanjingensis	α-Proteobacteria	*Wolbachia*	ES	[ai]
**Thripidae**				
Frankliniella occidentalis	γ-Proteobacteria	*Erwinia*	G/S	[a]
	γ-Proteobacteria	Bfo-1 (Erwinia close)	G/S	[d,i]
	γ-Proteobacteria	Bfo-2 (Pantoea close)	G/S	[d,i]
F. fusca	γ-Proteobacteria	*Pantoea ananatis*	G/S	[c,n]
	γ-Proteobacteria	*Pantoea agglomerans*	G/S	[c,n]

Aptinothrips rufus	α-Proteobacteria	*Wolbachia*	ES	h
Caliothrips fasciatus	α-Proteobacteria	*Wolbachia*	ES	f
Echinothrips americanus	α-Proteobacteria	*Wolbachia*	ES	e
Heliothrips haemorrhoidalis	α-Proteobacteria	*Wolbachia*	ES	k
Pezothrips kellyanus	α-Proteobacteria	*Wolbachia*	ES	m
	Bacteroidetes	*Cardinium*	ES	m
Sciothrips cardamomi	α-Proteobacteria	*Wolbachia*	ES	j
Scirtothrips dorsalis	189 genera , mainly Actinobacteria		G/S	g
	γ-Proteobacteria and α-Proteobacteria		G/S	g
Suocerathrips linguis	α-Proteobacteria	*Wolbachia*	ES	e
Thrips tabaci	γ-Proteobacteria	*Erwinia*	G/S	b
	γ-Proteobacteria	*Pantoea ananatis*	G/S	o
	γ-Proteobacteria	*Pantoea agglomerans*	G/S	o
T. palmi	α-Proteobacteria	*Wolbachia*	ES	p

[1]ES for intracellular endosymbionts; G/S for gut/saliva

[2]**References for Tetranychidae:** Breeuwer & Jacobs 1996[a]; Tsagarakou et al. 1996[b]; Breeuwer 1997[c]; Weeks & Breeuwer 2001[d]; Vala et al. 2002[e], 2004[f]; Gotoh et al. 2003[g], 2007[h]; Weeks et al. 2003[i]; Zchori-Fein & Perlman 2004[j]; Enigl et al. 2005[k]; Hoy & Jeyaprakash 2005[m]; Xie et al. 2006[n], 2016[o]; Enigl & Schausberger 2007[p]; Chen et al. 2009[q]; Nakamura et al. 2009[r]; Ros & Breeuwer 2009[s]; Ros et al. 2009[t], 2012[u]; Yoon et al. 2010[v]; Su et al. 2012[w]; Zhu et al. 2012[x], 2018[y]; Zindel 2012[z]; Zhang et al. 2013[aa], 2016[ab], 2018[ac]; Zhao et al. 2013a[ad]b[ae]; Choi et al. 2016[af]; Iskender & Aksu 2016[ag]; Knegt et al. 2017[ah] Sakamoto et al. 2017[ai]; Staudacher at al. 2017[aj]; Sato et al. 2018[ak]; Zele et al. 2018[am]

[3]**References for Thripidae:** De Vries et al. 2001[a], 2008[b]; Wells et al. 2002[c]; Chanbusarakum & Ullman 2008[d]; Kumm & Moritz 2008[e]; Rugman-Jones et al. 2012[f]; Dickey et al. 2014[g]; van der Kooi & Schwander 2014[h]; Facey et al. 2015[i]; Jacob et al. 2015[j]; Nguyen et al. 2015[k], 2017[m]; Dutta et al. 2016a[n]b[o]; Saurav et al. 2016[p]
