# Supplementary material for: Herbivore-Associated Bacteria as Potential Mediators and Modifiers of Induced Plant Defense Against Spider Mites and Thrips
Source: Front Plant Sci. 2018 Jul 30;9:1107. doi: 10.3389/fpls.2018.01107 (PMC6077224; doi:10.3389/fpls.2018.01107)
Supplement: Supplementary file 2 [file Table_2.pdf]

Table S2. References cited in Table S1.

Breeuwer, J. A. J. (1997). *Wolbachia* and cytoplasmic incompatibility in the spider mites *Tetranychus urticae* and *T. turkestani*. *Heredity* 79, 41-47.

Breeuwer, J. A. J., and Jacobs, G. (1996). *Wolbachia*: intracellular manipulators of mite reproduction. *Exp. Appl. Acarol.* 20, 421-434.

Chanbusarakum, L. and Ullman, D. (2008). Characterization of bacterial symbionts in *Frankliniella occidentalis* (Pergande), Western flower thrips. *J. Invert. Pathol.* 99, 318-325.

Chen, X. L., Xie, R. R., Li, G. Q., and Hong, X.-Y. (2009). Simultaneous detection of endosymbionts *Wolbachia* and *Cardinium* in spider mites (Acari: Tetranychidae) by multiplex-PCR. *Int. J. Acarol.* 35, 397-403.

Choi, O., Park, J. J., and Kim, J. (2016). *Tetranychus urticae* (Acari: Tetranychidae) transmits *Acidovorax citrulli*, causal agent of bacterial fruit blotch of watermelon. *Exp. Appl. Acarol*. 69, 445-451.

De Vries, E. J., Breeuwer, J. A.J., Jacobs, C., and Mollema, C. (2001). The association of western flower thrips, *Frankliniella occidentalis*, with a near *Erwinia* species gut bacteria: transient or permanent? *J. Invert. Pathol.* 77, 120-128.

De Vries, E. J., van der Wurfe, A. W. G., Jacobs, G., and Breeuwer, J. A. J. (2008). Onion thrips, *Thrips tabaci*, have gut bacteria that are closely related to the symbionts of the western flower thrips, *Frankliniella occidentalis. J. Insect Sci.* 8, 1-11.

Dickey, A. M., Trease, A. J., Jara-Cavieres, A., Kumar, V., Christenson, M. K., Potluri, L.-P., et al. (2014). Estimating bacterial diversity in *Scirtothrips dorsalis* Hood (Thysanoptera: Thripidae) via next generation sequencing. *Fla. Entomol.* 92, 362-366.

Dutta, B., Gitaitis, R, Barman, A., Avci, U., Marasigan, K., and Srinivasan, R. (2016a). Interactions between *Frankliniella fusca* and *Pantoea ananatis* in the center rot epidemic of onion (*Allium cepa*). *Phytopathol.* 106, 956-962.

Dutta, B., Barman, A., Srinivasan, R., Avci, U., Ullman, D.E., Langston, D.B., et al. (2016b). Transmission of *Pantoea ananatis* and *P. agglomerans,* causal agents of center rot of onion (*Allium cepa*), by onion thrips (*Thrips tabaci*) through feces. *Phytopathol.* 104, 812-819.

Enigl, M., and Schausberger, P. (2007). Incidence of the endosymbionts *Wolbachia*, *Cardinium* and *Spiroplasma* in phytoseiid mites and associated prey. *Exp. Appl. Acarol.* 42, 75-85.

Enigl, M., Zchori-Fein, E., and Schausberger, P. (2005). Negative evidence of *Wolbachia* in the predaceous mite *Phytoseiulus persimilis*. *Exp. Appl. Acarol.* 36, 249-262.

Facey, P. D., Meric, G., Hitchings, M. D., Pachebat, J. A., Hegarty, M. J., Chen, X., et al. (2015). Draft genomes, phylogenetic reconstruction, and comparative genomics of two novel cohabiting bacterial symbionts isolated from *Frankliniella occidentalis*. *Genome Biol. Evol.* 7, 2188-2202.

Gotoh, T., Noda, H., and Hong, X.-Y. (2003). *Wolbachia* distribution and cytoplasmic incompatibility based on a survey of 42 spider mite species (Acari: Tetranychidae) in Japan. *Heredity* 91, 208-216.

Gotoh, T., Noda, H., and Ito, S. (2007) *Cardinium* symbionts cause cytoplasmic incompatibility in spider mites. *Heredity* 98, 13-20.

Hoy, M. A., and Jeyaprakash, A. (2005). Microbial diversity in the predatory mite *Metaseiulus occidentalis* (Acari: Phytoseiidae) and its prey, *Tetranychus urticae* (Acari: Tetranychidae). *Biol. Control* 32, 427-441.

Iskender, N. A., and Aksu, Y. (2016). Isolation, characterization and pathogenicity of bacteria from *Oligonychus ununguis* (Jacobi) (Acari, Tetranychidae). *Fres. Environ. Bull.* 25, 4163-4170.

Jacob, T. K., D'Silva,S., Kumar, C. M. S., Devasahayam, S., Rajalakshmi, V., Suheeesh, E. S., et al. (2015). Single strain infection of adult and larval cardamom thrips (*Sciothrips cardamomi*) by *Wolbachia* subgroup Con belonging to supergroup B in India. *Invert. Reprod. Develop*. 59, 1-8.

Knegt, B., Potter, T., Pearson, N. A., Sato, Y., Staudacher, H., Schimmel, B. C. J. et al. (2017). Detection of genetic incompatibilities in non-model systems using simple genetic markers: hybrid breakdown in the haplodiploid spider mite *Tetranychus evansi*. *Heredity* 118, 311-321.

Kumm, S., and Moritz, G. (2008). First detection of *Wolbachia* in arrhenotokous populations of thrips species (Thysanoptera: Thripidae and Phlaeothripidae) and its role in reproduction. *Environ. Entomol*. 37, 1422-1428.

Nakamura, Y., Kawai, S., Yukuhiro, F., Ito, S., Gotoh, T., Kisimoto, R., et al. (2009). Prevalence of *Cardinium* bacteria in planthoppers and spider mites and taxonomic revision of "Candidatus *Cardinium hertigii*" based on detection of a new *Cardinium* group from biting midges. *Appl. Environ. Microbiol*. 75, 6757-6763.

Nguyen, D. T., Spooner-Hart, R. N., and Riegler, M. (2015). Polyploidy versus endosymbionts in obligately thelytokous thrips. *BMC Evol. Biol*. 15, 23.

Nguyen, D. T., Morrow, J. L., Spooner-Hart, R. N., and Riegler, M. (2017). Independent cytoplasmic incompatibility induced by *Cardinium* and *Wolbachia* maintains endosymbiont coinfections in haplodiploid thrips populations. *Evolution* 71, 995-1008.

Ros, V. I. D., and Breeuwer, J. A. J. (2009). The effects of, and interactions between, *Cardinium* and *Wolbachia* in the doubly infected spider mite *Bryobia sarothamni*. *Heredity* 102, 413-422.

Ros, V. I. D., Fleming, V. M., Feil, E. J., and Breeuwer, J. A. J. (2009). How diverse is the genus *Wolbachia*? Multiple-gene sequencing reveals a putatively new *Wolbachia* supergroup recovered from spider mites (Acari: Tetranychidae). *Appl. Environ. Microbiol*. 75, 1036-1043.

Ros, V. I. D., Fleming, V. M., Feil, E. J., and Breeuwer, J. A. J. (2012). Diversity and recombination in *Wolbachia* and *Cardinium* from *Bryobia* spider mites. *BMC Microbiol.* 12, S13.

Rugman-Jones, P. F., Hoddle, M. S., Mound, L. A., and Stouthamer, R. (2006). Molecular identification key for pest species of *Scirtothrips* (Thysanoptera: Thripidae). *J. Econ. Entomol*. 99, 1813-1819.

Sakamoto, H., Matsuda, T., Suzuki, R., Saito, Y., Lin, J.-Z., Zhang, Y.-X., et al. (2017). Molecular identification of seven species of the genus *Stigmaeopsis* (Acari: Tetranychidae) and preliminary attempts to establish their phylogenetic relationship. *Syst. Appl. Acarol.* 22, 91-101.

Sato, Y., Sakamoto, H., Gotoh, T., Saito, Y., Chao, J.-T., Egas, M. et al. (2018). Patterns of reproductive isolation in a haplodiploid – strong post-mating, prezygotic barriers among three forms of a social spider mite. *J. Evol. Biol.* 31, 866-881.

Saurav, G. K., Daimei, G., Rana, V.S., Popli, S., and Rajagopal, R. (2016). Detection and localization of *Wolbachia* in *Thrips palmi* Karny (Thysanoptera: Thripidae). *Indian J. Microbiol*. 56, 167-171.

Su, H. H., Jiang, F., Yu, M. Z., Yang, X. M., Yang, Y. Z., and Hong, X. Y. (2012). Effects of *Wolbachia* on rDNAITS2 variation and evolution in natural populations of *Tetranychus urticae* Koch. *Syst. Appl. Acarol*. 17, 45-52.

Tsagkarakou, A., Guillemaud, T., Rousset, F., and Navajas, M. (1996). Molecular identification of a *Wolbachia* endosymbiont in a *Tetranychus urticae* strain. *Insect. Mol. Biol.* 5, 217-221.

Vala, F., Egas, M., Breeuwer, J. A. J., and Sabelis, M. W. (2004). *Wolbachia* affects oviposition and mating behaviour of its spider mite host. *J. Evol. Biol.* 17, 692-700.

Vala, F., Weeks, A., Claessen, D., Breeuwer, J. A. J., and Sabelis, M. W. (2002). Within- and between population variation for *Wolbachia*-induced reproductive incompatibility in a haplodiploid mite. *Evolution* 56, 1331-1339.

Van der Kooi, C. J. and Schwander, T. (2014). Evolution of asexuality via different mechanisms in grass thrips (Thysanoptera: *Aptinothrips*). *Evolution* 68, 1883-1893.

Weeks, A. R. and Breeuwer, J. A. J. (2001). *Wolbachia*-induced parthenogenesis in a genus of phytophagous mites. *Proc. R. Soc. B.-Biol. Sci*. 268, 2245-2251.

Weeks, A. R., Velten, R., and Stouthamer, R. (2003). Incidence of a new sex-ratio-distorting endosymbiotic bacterium among arthropods. *Proc. R. Soc. B-Biol. Sci.* 270, 1857-1865.

Wells, M. l., Gitaitis, R. D., and Sanders, F. H. (2002). Association of tobacco thrips, *Frankliniella fusca* (Thysanoptera: Thripidae) with two species of bacteria of the genus *Pantoea. Ann. Entomol. Soc. Am.* 95, 719-723.

Xie, L., Miao, H., and Hong, X.-Y. (2006). The two-spotted spider mite *Tetranychus urticae* Koch and the carmine spider mite *Tetranychus cinnabarinus* (Boisduval) in China mixed in their *Wolbachia* phylogenetic tree. Zootaxa 1165, 33-46.

Xie, R. R., Sun, J.-T., Xue, X. F., and Hong, X.-Y. (2016). Cytoplasmic incompatibility and fitness benefits in the two-spotted spider mite *Tetranychus urticae* (red form) doubly infected with *Wolbachia* and *Cardinium*. *Syst. Appl. Acarol*. 21, 1161-1173.

Yoon, C., Indiragandhi, P., Anandham, R., Cho, S., Sa, T. M., and Kim, G. H. (2010). Bacterial diversity and distribution from the whole mite extracts in acaricide resistant and susceptible populations of twospotted spider mite *Tetranychus urticae* (Acari: Tetranychidae). *J. Kor. Soc. App. Biol. Chem*. 53, 446-457.

Zélé, F., Santos, I., Olivieri, I., Weill, M., Duron, O., and Magalhães, S. (2018). Endosymbiont diversity and prevalence in herbivorous spider mite populations in South-Western Europe. *FEMS Microb. Ecol.* 94, fiy015.

Zchori-Fein, E., and Perlman, S. J. (2004). Distribution of the bacterial symbiont *Cardinium* in arthropods. *Mol. Ecol*. 13, 2009-2016.

Zhang, Y. K., Chen, Y. T., Yang, K., Qiao, G. X., and Hong, X.-Y. (2016). Screening of spider mites (Acari: Tetranychidae) for reproductive endosymbionts reveals links between co-infection and evolutionary history. *Sci. Rep*. 6, 27900.

Zhang, Y. K., Yang, K., Zhu, Y.-X., and Hong, X.-Y. (2018). Symbiont-conferred reproduction and fitness benefits can favour their host occurrence. *Insect Sci.* 8, 1626-1623.

Zhang, Y. K., Zhang, K. J., Sun, J. T., Yang, X. M., Ge, C., and Hong, X.-Y. (2013). Diversity of *Wolbachia* in natural populations of spider mites (genus *Tetranychus*): Evidence for complex infection history and disequilibrium distribution. *Microb. Ecol*. 65, 731-739.

Zhao, D. X., Chen, D. S., Ge, C., Gotoh, T., and Hong, X.-Y. (2013a). Multiple infections with *Cardinium* and two strains of *Wolbachia* in the spider mite *Tetranychus phaselus* Ehara: Revealing new forces driving the spread of *Wolbachia*. *PLoS ONE* 8, e54964.

Zhao, D. X., Zhang, X. F., and Hong, X.-Y. (2013b). Host-symbionts interactions in spider mite *Tetranychus truncatus* doubly infected with *Wolbachia* and *Cardinium*. *Environ. Entomol*. 42, 445-452.

Zhu, L. Y., Zhang, K. J., Zhang, Y. K., Ge, C., Gotoh, T., and Hong, X.-Y. (2012). *Wolbachia* strengthens *Cardinium*-induced cytoplasmic incompatibility in the spider mite *Tetranychus piercei* McGregor. *Curr. Microbiol.* 65, 516-523.

Zhu, Y.-X., Song, Y.-L., Zhang, Y.-K., Hoffmann, A. A., Zhou, J.-C., Sun, J.-T., et al. (2018) Incidence of facultative bacterial endosymbionts in spider mites associated with local environment and host plant. *Appl. Environ. Microbiol.* 84, e02546-17.

Zindel, S. (2012). *Mites and endosymbionts – towards improved biological control*. PhD thesis University of Neuchatel, Switzerland, 156 pp.
